# Platelet size for distinguishing between inherited thrombocytopenias and immune thrombocytopenia: a multicentric, real life study

**DOI:** 10.1111/bjh.12349

**Published:** 2013-04-25

**Authors:** Patrizia Noris, Catherine Klersy, Paolo Gresele, Fiorina Giona, Paola Giordano, Pietro Minuz, Giuseppe Loffredo, Alessandro Pecci, Federica Melazzini, Elisa Civaschi, Annamaria Mezzasoma, Monica Piedimonte, Fabrizio Semeraro, Dino Veneri, Francesco Menna, Laura Ciardelli, Carlo L Balduini, on behalf of the Italian ‘Gruppo di Studio delle Piastrine’

**Affiliations:** 1 Department of Internal Medicine, Clinical Chemistry Laboratory, Biometry and Clinical Epidemiology Service, University of Pavia and IRCCS Policlinico San Matteo FoundationPavia, Italy; 2 Department of Internal Medicine, Division of Internal and Cardiovascular Medicine, University of PerugiaPerugia, Italy; 3 Department of Cellular Biotechnologies and Haematology, Sapienza UniversityRome, Italy; 4 Department of Biomedical Sciences and Human Oncology, University of BariBari, Italy; 5 Department of Medicine and Haematology, University Hospital of VeronaVerona, Italy; 6 Department of Oncology, Azienda ‘Santobono-Pausilipon’, Pausilipon HospitalNaples, Italy

**Keywords:** inherited thrombocytopenias, immune thrombocytopenia, platelet volume, platelet diameter, platelet size

## Abstract

The most frequent forms of inherited thrombocytopenia (IT) are characterized by platelet size abnormalities and it has been suggested that this parameter is useful for their differentiation from immune thrombocytopenia (ITP). Recently, a monocentric study identified cut-off values for mean platelet volume (MPV) and mean platelet diameter (MPD) with good diagnostic accuracy in this respect. To validate these cut-off values in a different and larger case series of patients, we enrolled 130 subjects with ITP and 113 with IT in six different centres. The platelet count and MPV was each measured by the instrument routinely used in each institution. In some centres, platelet count was also measured by optical microscopy. MPD was evaluated centrally by image analysis of peripheral blood films. The previously identified cut-off value for MPV had 91% specificity in distinguishing ITP from inherited macrothrombocytopenias (mono and biallelic Bernard-Soulier, *MYH9*-related disease), while its sensitivity was greatly variable depending on the instrument used. With an appropriate instrument, specificity was 83%. The diagnostic accuracy of MPD was lower than that obtained with MPV. We concluded that MPV is a useful parameter for differentiating ITP from IT provided that it is measured by appropriate cell counters.

Distinguishing immune thrombocytopenia (ITP) from inherited thrombocytopenias (ITs) is not always easy, and several patients with inherited forms have received unnecessary medical treatments or even splenectomy because they were misdiagnosed with ITP (Bader-Meunier *et al*, 2003; Noris *et al*, 2004, 2012; Kunishima *et al*, 2006; Gunay-Aygun *et al*, 2010; Savoia *et al*, 2011). Differential diagnosis may be especially difficult when a low platelet count, which can be used to decide whether thrombocytopenia is acquired or congenital, is incidentally discovered in asymptomatic subjects who have never had a blood count performed before. In some cases, family history may solve the diagnostic dilemma, in that the presence of other relatives with low platelet counts strongly argues against ITP and supports an inherited form. Conversely, the absence of affected family members by no means excludes genetic disorders because many ITs are transmitted in a recessive fashion or derive from *de novo* mutations.

As many forms of IT are characterized by platelet macrocytosis (Balduini & Savoia, 2012), it is commonly accepted that the evaluation of platelet size is an important tool to provoke suspicion of these disorders. However, the accurate measurement of platelet size in IT subjects presents notable difficulties because some of the less rare forms, such as *MYH9*-related disease (*MYH9*-RD), monoallelic Bernard-Soulier syndrome (BSS) and biallelic BSS, may present platelets that, due to their very large size, are not recognized by the electronic counters, which therefore underestimates not only the platelet count but also the mean platelet volume (MPV) (Balduini *et al*, 2011; Savoia *et al*, 2011; Noris *et al*, 2012). Moreover, instruments operating on different principles also induce variability into the measurement of MPV in healthy subjects or in subjects with non-macrocytic thrombocytopenias, thus making difficult the direct comparison of MPV values obtained in different centres (Latger-Cannard *et al*, 2012).

A possible alternative for the evaluation of platelet size is microscopic examination of peripheral blood smears, which recognizes platelets regardless of their size and thus makes possible accurate measurement of their diameters (Noris *et al*, 2009). However, this method also has drawbacks in that it is time consuming and requires experienced operators. Finally, a major problem for the use of platelet size in differentiating ITs from ITP was represented until recently by the lack of cut-off values for both MPV and mean platelet diameter (MPD).

To remedy the last difficulty, a recent, monocentric study measured platelet size in patients with inherited macrothrombocytopenias and ITP by both cell counters and optical microscopy on blood films. It found that both techniques were effective in distinguishing the two conditions, in that a MPV higher than 12·4 fl measured with an optical cell counter (ADVIA 120) and a MPD larger than 3·3 μm had sensitivity and specificity higher than 80% (Noris *et al*, 2009).

To validate these cut-off values, we performed a multicentre study in a different series of patients, which enrolled 113 consecutive patients with ITs and 130 patients with ITP.

## Patients and methods

### Patients

All of the patients with ITs or ITP examined in the six clinical centres involved in this project between January 2010 and January 2012 were enrolled in the study. All of them were Italian. Their main characteristics are reported in Table 1. Diagnosis of ITP was made according to the recommendations of the ‘International consensus report on the investigation and management of primary immune thrombocytopenia’ (Provan *et al*, 2010). The diagnosis of IT was made in subjects who had been thrombocytopenic since birth with no identifiable cause of low platelet count and in cases where thrombocytopenia was clearly transmitted in a hereditary manner. The search for specific forms of inherited thrombocytopenias was made by the diagnostic algorithm of the Italian ‘Gruppo di Studio delle Piastrine’ (Platelet Study Group) (Balduini *et al*, 2003; Noris *et al*, 2004). Patients with an IT that did not fit the criteria for any known genetic disorder were classified as affected by a form of unknown origin.

**Table 1:** Main characteristics of patients with different disorders evaluated in each centre.

	Centre 1			Centre 2			Centre 3			Centre 4			Centre 5			Centre 6		
	Patients (*N*)	Age (years), mean (25th–75th)	F/M	Patients (*N*)	Age (years), mean (25th–75th)	F/M	Patients (*N*)	Age (years), mean (25th–75th)	F/M	Patients (*N*)	Age (years), mean (25th–75th)	F/M	Patients (*N*)	Age (years), mean (25th–75th)	F/M	Patients (*N*)	Age (years), mean (25th–75th)	F/M
*MYH9*-RD	17	34 (20–45)	11/6	13	39 (22–54)	5/8	0	–	–	2	11 (2–20)	2/0	0	–	–	0	–	–
Biallelic BSS	2	33 (32–34)	0/2	0	–	–	0	–	–	0	–	–	0	–	–	0	–	–
Monoallelic BSS	11	43 (22–70)	3/8	1	12	0/1	0	–	–	0	–	–	0	–	–	0	–	–
*ITGA2B*/*ITGB3*-RT	2	36 (34–39)	2/0	1	38	1/0	0	–	–	0	–	–	0	–	–	0	–	–
*ANKRD26*-RT	34	38 (25–54)	14/20	0	–	–	3	19 (11–26)	2/1	0	–	–	0	–	–	0	–	–
Unknown IT	12	36 (18–51)	4/8	2	31 (24–38)	1/1	9	15 (3–14)	3/6	1	18	0/1	3	6 (2–8)	0/3	0	–	–
ITP	36	43 (26–57)	22/14	14	46 (29–64)	7/7	26	11 (9–13)	14/12	7	12 (10–16)	3/4	27	15 (11–19)	17/10	20	56 (34–70)	13/7

F, female; M, male; IT, inherited thrombocytopenia; ITP, immune thrombocytopenia.

This study was approved by the ethical committee of each clinical institution and patients gave informed consent to participate.

### Platelet analyses

Platelet counts and MPVs were obtained by the cell counters used routinely in each centre without any specific recommendation to the laboratory technicians performing the test. Analyses were executed within 3 h after sampling. The instruments used in different centres were as follows:

Centre 1: Cell-Dyn 3700 (Abbott, Lake Forest, IL, USA), using the optical channel of the instrument;
Centre 2: HeCo 5 (Radim-Seac, Florence, Italy), which analyses platelets by an impedentiometric method;
Centre 3: This centre initially used an ADVIA 2120 (Siemens, Tarrytown, NY, USA), which used the optical method, then a Cell-Dyn 3700 (Abbott), using the impedance channel of the instrument;
Centre 4: XE-2100 (Sysmex Corporation, Kobe, Japan) (impedance method);
Centre 5: ADVIA 2120i (Siemens, optical method);
Centre 6: ADVIA 120 (Siemens, optical method).

As it is well known that MPV varies according to the cell counter used, the value measured in each subject was expressed not only as an absolute value, but also as % difference with respect to the median value obtained in 30 healthy subjects analysed at each centre with the same instrument. To verify the consistency of our findings, the same analyses were performed by using the Z-score, with similar results (data not shown). In the earlier study that determined the best cut-off value of MPV to be 12·4 fl for differentiating inherited macrothrombocytopenias from ITP, the median MPV of control subjects was 8·2 fl (Noris *et al*, 2009). Thus, this cut-off value was transformed in an increase of more than 51% with respect to the median value in controls.

Whenever available, the platelet count was also measured by optical microscopy (Noris *et al*, 2009). EDTA-anticoagulated blood was diluted in ammonium oxalate solution and the counting procedure was performed in a Neubauer chamber, as indicated by the International Committee for Standardization in Haematology (England *et al*, 1988).

The assessment of MPD was centralized. Blood smears were prepared in each centre from blood obtained by finger stick and were shipped to the centre that performed the test. Here, blood films were stained with May–Grünwald–Giemsa and the largest diameter of each platelet was measured by optical microscopy with software-assisted image analysis (Axio-vision 4.5; Carl Zeiss, Göttingen, Germany). For each subject, the MPD was the median value obtained in 200 cell measurements. To identify the normal values of this parameter, MPD was measured in 50 healthy subjects.

### Statistical analysis

Descriptive statistics were computed as median and 25th–75th percentiles for continuous variables and as counts and % for categorical variables. The Kruskall–Wallis test was used to compare MPV (transformed, see above) and MPD between diagnostic groups. The diagnostic accuracy (sensitivity, specificity and predictive values, and 95% confidence intervals [95% CI]) to distinguish inherited macrothrombocytopenia from ITP according to MPD (cut-off >3·3 μm) and MPV (cut-off >51%) was assessed. Receiver-operated characteristic (ROC) curve analysis was used to identify optimal cut-offs for MPV and MPD and selected diagnostic groups.

Stata 12.1 (StataCorp, College Station, TX, USA) and Medcalc 12.3 (MedCalc Software, Broekstraat 52, 9030 Mariakerke, Belgium) were used for computation. A 2-sided *P*-value <0·05 was considered statistically significant. The Bonferroni correction was used for *post-hoc* comparisons.

## Results

### Patients

Two hundred and forty-three patients were enrolled in the study. One hundred and thirty were affected by primary ITP in any phase of the disease (newly diagnosed *n* = 25, persistent ITP *n* = 20 and chronic ITP *n* = 85) and 113 by ITs. In particular, 32 subjects had *MYH9*-RD (Balduini *et al*, 2011), two had biallelic BSS (Savoia *et al*, 2011), 12 had monoalleic BSS due to Bolzano mutation in *GP1BA* (Noris *et al*, 2012), 37 had a form of autosomal dominant thrombocytopenia deriving from *ANKRD26* mutations (*ANKRD26*-RT) (Noris *et al*, 2011), and three an autosomal dominant thrombocytopenia due to an integrin beta3 mutation (*ITGA2B*/*ITGB3*-RT) (Gresele *et al*, 2009). All these diagnoses were confirmed by the identification of the causative mutations. *MYH9*-RD, mono and biallelic BSS and *ITGA2B*/*ITGB3*-RT are currently classified as inherited macrothrombocytopenias, while *ANKRD26*-RT has been reported to have platelets of normal size (Balduini *et al*, 2011).

Twenty-seven patients did not fit the criteria for any known disorder and were classified as affected by an IT of unknown origin. The number of patients with ITs was higher than expected compared to ITP, but this largely derived from a bias in enrolment because two centres involved in the study are particularly interested in ITs both in terms of clinical care and scientific research. Thus, the composition of this case series cannot be used for epidemiological considerations.

### Platelet count

Median platelet counts measured by electronic counters in different categories of patients are reported in Table 2, together with the available values obtained by microscope counting. As it is well known that microscope counting is largely operator-dependent, the differences between the results obtained with the two methods are not surprising. However, in most cases of inherited macrothrombocytopenias, platelet counts measured by the counters were lower than those measured by microscope. In some cases, as in the patients with *MYH9*-RD investigated in Centre 2 and subjects with biallelic BSS evaluated in Centre 1, discrepancies between the two methodologies were very large. Given that *MYH9*-RD and biallelic BSS are characterized by the presence of very large platelets, a possible explanation for these discrepancies is that the electronic counters underestimated platelet count because they did not recognize the largest elements. As very large platelets can also be observed in ITP (see below), the same phenomenon could explain the much higher platelet counts obtained by microscope methodology in patients with this disorder examined in Centres 2 and 4.

**Table 2:** Platelet counts in patients and controls. In Centre 3, the cell counter was replaced during the study and the median MPVs obtained by the two instruments (ADVIA 2120 and Cell-Dyn 3700) in patients and controls are reported separately

	Centre 1		Centre 2		Centre 3			Centre 4		Centre 5		Centre 6	
						
	Platelet count × 10^9^/l, median (25th–75th)		Platelet count × 10^9^/l, median (25th–75th)		Platelet count × 10^9^/l, median (25th–75th)			Platelet count × 10^9^/l, median (25th–75th)		Platelet count × 10^9^/l, median (25th–75th)		Platelet count × 10^9^/l, median (25th–75th)	
	
	Counter	Manual	Counter	Manual	Counter (ADVIA)	Counter (Cell-Dyn)	Manual	Counter	Manual	Counter	Manual	Counter	Manual
*MYH9*-RD	47 (19–55)	56 (34–75)	33 (19–41)	62 (35–77)	–	–		73 (65–82)	–	–	–	–	–
Biallelic BSS	12 (6–18)	45 (38–52)	–	–	–	–		–	–	–	–	–	–
Monoallelic BSS	115 (94–139)	116 (68–144)	20	20	–	–		–	–	–	–	–	–
*ITGA2B*/*ITGB3*-RT	82 (48–116)	85 (56–114)	61	106	–	–		–	–	–	–	–	–
*ANKRD26*-RT	52 (29–73)	49 (21–62)	–	–	34 (14–70)	–		–	–	–	–	–	–
Unknown IT	112 (100–132)	106 (98–121)	85 (50–120)	120 (60–140)	77 (18–109)	80 (56–104)	80 (68–92)	135	148	96 (47–144)	–	–	–
ITP	40 (21–59)	44 (23–58)	71 (53–94)	97 (70–110)	50 (25–63)	42 (26–50)	41 (28–56)	60 (17–77)	85 (74–97)	66 (28–96)	–	55 (33–81)	–

IT, inherited thrombocytopenia; ITP, immune thrombocytopenia.

### Platelet volume and platelet diameter

Table 3 reports the medians of the values of MPV and MPD measured in patients with different categories of thrombocytopenias examined in each centre as well as in controls. Concerning MPV, it is important to note that the two impedentiometric counters used in Centres 3 and 4 did not give the MPV values for 10 of 20 patients with ITP and in a few cases of *MYH9*-RD or IT of unknown origin. Failure of impedentiometric instruments in measuring MPV in a large percentage of subjects with enlarged platelets is not new and has been attributed to abnormalities of platelet distribution curves (Noris *et al*, 2009).

**Table 3:** Medians of MPV and MPD of patients investigated in different centres. Two values of MPV are reported for Centre 3 because two cell counters were used (ADVIA 2120 and Cell-Dyn 3700)

	Centre 1		Centre 2		Centre 3			Centre 4		Centre 5		Centre 6	
					
	MPV (fl), median (25th–75th)	MPD (μm), median (25th–75th)	MPV (fl), median (25th–75th)	MPD (μm), median (25th–75th)	MPV (fl) ADVIA, median (25th–75th)	MPV (fl) Cell-Dyn, median (25th–75th)	MPD (μm), median (25th–75th)	MPV (fl), median (25th–75th)	MPD (μm), median (25th–75th)	MPV (fl), median (25th–75th)	MPD (μm), median (25th–75th)	MPV (fl), median (25th–75th)	MPD (μm), median (25th–75th)
*MYH9*-RD	21·2 (19·9–23·8)	4·2 (3·8–4·5)	13·3 (12·6–13·7)	4·4 (3·4–5·2)	–	–	–	‡	3·2 (3·0–3·3)	–	–	–	–
Biallelic BSS	16·6 (16·4–16·9)	4·1 (4–4·2)	–	–	–	–	–	–	–	–	–	–	–
Monoallelic BSS	15·5 (14·6–16·7)	3·4 (3–3·9)	15·1	2·8	–	–	–	–	–	–	–	–	–
*ITGA2B*/*ITGB3*-RT	11·6 (10·2–13·0)	3·5 (3·3–3·6)	14·8	3·7	–	–	–	–	–	–	–	–	–
*ANKRD26*-RT	8·4 (7·9–9)	2·8 (2·5–3)	–	–	7·6 (6·3–8·5)	–	2·6 (2·2–2·9)	–	–	–	–	–	–
Unknown IT	11·6 (9·6–13·4)	3 (2·8–3·2)	10·3 (10·3–10·4)	3·1 (3–3·2)	11·8 (9·4–13·3)	6·2*	2·6 (2·2–2·9)	10·7	2·5	9 (7–11)	2·7 (2–3·1)	–	–
ITP	11·6 (9·7–12·7)	3·1 (2·7–3·4)	13·2 (12·2–14·6)	3 (2·7–3·2)	10·3 (9–10·9)	12·5† (10·4–15)	2·9 (2·6–3·2)	12·3§ (11·6–12·9)	2·8 (2·5–3)	9·7 (8·1–11·2)	3·2 (2·9–3·5)	10·5 (10–11·6)	3·2 (2·8–3·5)
Controls	8·8 (8·1–9·4)	2·4 (2·2–2·6)	10·9 (10·3–11·5)	2·4 (2·2–2·6)	8·0 (7·5–8·4)	8·8 (7·8–9·6)	2·4 (2·2–2·6)	10·0 (8·5–10·8)	2·4 (2·2–2·6)	7·4 (6·8–7·8)	2·4 (2·2–2·6)	8·2 (7·4–8·9)	2·4 (2·2–2·6)

* MPV not given by the counter in 1 of 2 patients.

† Not given in 7 of 13 patients.

‡ Not given in 2 of 2 patients.

§ Not given in 3 of 7 patients.

IT, inherited thrombocytopenia; ITP, immune thrombocytopenia.

Another interesting observation is that the MPV of *MYH9*-RD patients was much higher in Centre 1 than in Centre 2 although MPD was quite similar. Together with the already discussed discrepancies between platelet counts obtained in Centre 2 by cell counter and microscope, this observation strongly supports the hypothesis that the impedentiometric instrument of this institution had major problems in recognizing large platelets of *MYH9*-RD and therefore greatly underestimated both platelet count and platelet volume.

**Fig 1:**
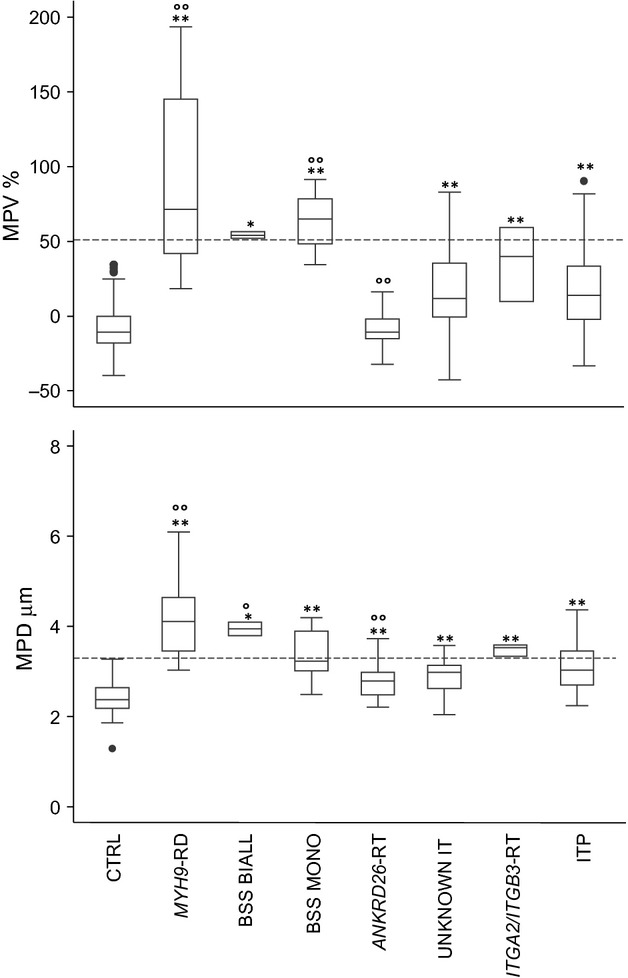
Mean platelet volume (MPV) and mean platelet diameter (MPD) in different categories of subjects. To allow comparison of the values obtained with different cell counters, MPV was expressed as median % difference with respect to the median MPV observed in controls. Medians (line within the boxes), standard deviation (boxes), 25th–75th percentiles (boxes), non-outlier extremes (whiskers) and outliers (dots) are reported. The cut-off values for distinguishing immune thrombocytopenia (ITP) from *MYH9*-RD, mono- and biallelic-Bernard-Soulier syndrome (BSS) are indicated by dashed lines. Kruskall Wallis test: *P* < 0·001 for both MPV and MPD comparisons between groups. * *P* < 0·05, ** *P* < 0·01 with respect to control; ° *P* < 0·05, °° *P* < 0·01 with respect to ITP, after Bonferroni correction.

The median MPV, expressed as % with respect to the median value of healthy subjects, was significantly higher than controls in *MYH9*-RD, monoallelic and biallelic BSS, *ITGA2B*/*ITGB3*-RT and ITs of unknown origin (Fig 1). MPV was higher than control also in ITP, where similar values have been observed in all stages of the disease (data not shown). In contrast, the MPV of patients with *ANKRD26*-RT was not different from that obtained in healthy subjects. These results confirm that platelets have normal volume in *ANKRD26*-RT (Noris *et al*, 2011) and substantiate the current classification of *MYH9*-RD, BSS and *ITGA2B*/*ITGB3*-RT as macrothrombocytopenias (Balduini *et al*, 2011). Moreover, it is important to reiterate that the cell counter used by Centre 2 greatly underestimated MPV in *MYH9*-RD. On this basis, we suggest that the actual MPV in this disorder is higher than that reported in Fig 1 and that the value obtained in Centre 1 (249% of controls, see Table 3) better describes MPV in *MYH9*-RD.

The MPV of patients with *MYH9*-RD and monoallelic BSS was significantly higher also with respect to ITP. Conversely, the average MPV in ITP was not significantly different from those measured in biallelic BSS, ITs of unknown origin and *ITGA2B*/*ITGB3*-RT, while it was significantly higher than in *ANKRD26*-RT. Despite these statistically significant differences, the values obtained in all ITs largely overlapped those observed in ITP.

The median values of MPD in patients enrolled in each centre are reported in Table 3 together with the values obtained in controls. The median values in different categories of patients and controls are reported in Fig 1. Platelet diameters obtained in each category of subjects enrolled in different centres were similar, and this observation further supports the hypothesis that the observed variability of MPV was dependent on the cell counters used. As for MPV, the MPD was also higher than controls in patients with *MYH9*-RD, monoallelic and biallelic BSS, *ITGA2B*/*ITGB3*-RT, ITs of unknown origin and ITP. Instead, in contrast to MPV, MPD was also significantly higher than controls in *ANKRD26*-RT.

As shown in Fig 1, MPD was significantly higher than ITP in *MYH9*-RD and biallelic BSS, while it was lower than ITP in *ANKRD26*-RT. Of note, as for MPV, the variability of platelet diameters within each category of patients was large. As the measure of MPDs was centralized, we can exclude that technical differences played an important role in this variability, which, therefore has to be considered actual. Investigating the role of differences in genetic background, causative mutations, age or sex in this variability requires a very large number of patients for each disorder and, therefore, was not an aim of our study.

### Specificity and sensibility of the previously identified cut-off values of MPV and MPD in differentiating inherited thrombocytopenias from ITP

The already discussed cut-off values of MPV and MPD for distinguishing between inherited macrothrombocytopenias and ITP were identified by the study of subjects with *MYH9*-RD and monoallelic or biallelic BSS, in addition to ITP subjects (Noris *et al*, 2009). Thus, to confirm their effectiveness, we applied them to the patients of our case series with the same disorders.

The limit for MPV of more than 51% with respect to controls had 91·5% specificity (95% CI 84·8–95·8) and 65·9% (95% CI 50·1–79·5) sensitivity in distinguishing the above disorders from ITP. Based on the observed prevalence, the positive predictive value was 74·4% (95% CI 57·9–87) and the negative predictive value was 87·7% (95% CI 80·5–93). To correctly interpret these findings it is necessary to note that they derived from data obtained by various cell counters with different ability to recognize abnormally large platelets. We suggest that the 91·5% specificity emerging from global analysis is overestimated because the impedentiometric counters used in Centres 3 and 4 did not give the MPV in 10 ITP patients, in all likelihood due to the presence of very large platelets. We also suggest that the 65·9% sensitivity obtained in the global analysis underestimates the potential usefulness of MPV in differentiating inherited macrothrombocytopenias from ITP. As already discussed, the impedentiometric counter used in Centre 2 had a major problem in recognizing very large platelets and, as a consequence, none of the patients with inherited macrothrombocytopenias had a MPV higher than 51% of controls (sensitivity = 0%). Conversely, 25 of the 30 subjects with inherited macrothrombocytopenias examined in Centre 1 had MPVs higher than the cut-off value and the sensitivity of the test was 83·3% (95% CI 65·3–94·4).

As already reported, patients with *ITGA2B*/*ITGB3*-RT or an IT of unknown origin had MPV similar to ITP. Thus, the examined cut-off value for MPV has no role in differentiating these disorders. However, since MPV was significantly lower in *ANKRD26*-RT than in ITP, we searched by ROC analysis the ‘optimal’ cut-off value for discriminating between these conditions and measured its predictive value. Results of this analysis revealed that the best cut-off value was a MPV lower by more than 8% with respect to controls and that it had 54·1% sensitivity (95% CI 36·9–70·5) and 82·1% specificity (95% CI 73·9–88·5) in distinguishing *ANKRD26*-RT from ITP.

Concerning the cut-off value of 3·3 μm for MPD, it had 66·9% (95% CI 58·1–74·9) specificity and 75·5% (95% CI 61·1–86·7) sensitivity in differentiating *MYH9*-RD and monoallelic or biallelic BSS from ITP. Positive predictive value was 46·3% (95% CI 35–57·8) and negative predictive value 87·9 (95% CI 79·8–93·6). As for MPV, also MPD was lower in *ANKRD26*-RT than in ITP and we therefore searched for the best cut-off value for differentiating these conditions. We found that a platelet diameter of less than 3·0 μm had 78·4% sensitivity and 53·1% specificity in distinguishing *ANKRD26*-RT from ITP.

## Discussion

Platelet size is enlarged in many forms of IT and diagnostic guidelines suggest that this parameter is an important element for distinguishing these conditions from ITP (Geddis & Balduini, 2007; Provan *et al*, 2010). However, this statement is mainly based on expert opinion, because only one experimental study has addressed this matter in detail to date (Noris *et al*, 2009). In a single institution case series of patients with inherited macrothrombocytopenias (*MYH9*-RD, mono and biallelic BSS) and ITP, the authors measured MPDs by peripheral blood film evaluation and MPVs by two different blood cell analysers (ADVIA 120 [Bayer, Leverkusen, Germany] that uses two-dimensional light scatter and XE-2100 [Sysmex XE] based on the impedance method), and searched for the best cut-off values for distinguishing between ITP and inherited disorders. Results of this study revealed that both cell counters underestimated platelet counts in inherited macrothrombocytopenias with respect to the values obtained by microscope counting in a Neubauer chamber, although this phenomenon was much more evident with the impedentiometric counter (Noris *et al*, 2009). Moreover, the impedance instrument did not report the MPV in all the patients with inherited macrothrombocytopenia owing to the abnormalities in their platelet volume distribution curves. Finally, 3·3 μm for MPD and 12·4 fl for MPV measured by the optical counter were identified by ROC analysis as the best cut-off values for differentiating ITP from inherited macrothrombocytopenias. Sensitivity and specificity of MPV were 83% and 89% respectively, while those of MPD were 89% and 88%. Thus, Noris *et al* (2009) first demonstrated the effectiveness of platelet size in distinguishing between these conditions.

The present study aimed at further investigating this matter in a new, larger series of consecutive patients with ITP and various forms of IT evaluated in different centres. While evaluation of MPD was centralized, MPV was measured in each institution by the haematology analyser used routinely without any particular recommendation (‘real life’ study).

The results we obtained confirm and extend many of the findings of previous investigation, but also challenge some previous conclusions. First of all, we brought new evidence supporting the inadequacy of the haematological analysers that rely on impedance in measuring platelet parameters of subjects with enlarged platelets. Major problems in this regard have been reported previously with the Sysmex XE-2100 and the Beckman Coulter LH750 (Noris *et al*, 2009; Latger-Cannard *et al*, 2012), and similar limitations have been observed in the present study with the Sysmex XE-2100 used in Centre 4 and with the impedentiometric channel of the Abbott Cell-Dyn 3700 instrument used in Centre 3: both counters did not measure MPV in several patients with *MYH9*-RD, ITs of unknown origin or ITP because of their inability to recognize very large platelets. Moreover, the Radim-Seac HeCo 5 analyser used in Centre 2 greatly and systematically underestimated platelet count and MPV in subjects with *MYH9*-RD, who typically have giant platelets. Conversely, the Siemens ADVIA 2120 and 120, used in Centres 5 and 6 respectively, to study patients with ITP or ITs of unknown origin, measured platelet parameters in all examined cases and gave values of MPV that did not conflict with platelet diameters evaluated on peripheral blood films. The ADVIA 120 instrument was previously used in study reported by Noris *et al* (2009), which evaluated platelet parameters in inherited macrothrombocytopenias and ITP and proved to be more effective than the Sysmex XE-2100 used in parallel, even if it did not recognize platelets with an extreme degree of platelet macrocytosis. Finally, the optical channel of the Abbott Cell-Dyn 3700, which was utilized in Centre 1 to evaluate all the categories of subjects included in the study, gave platelet counts and MPVs that did not contrast with those measured by optical microscopy, with the only exception of the two examined patients with biallelic BSS, who had their platelet counts and MPVs greatly underestimated. Thus, the present data and those from the literature indicate that all cell counters have problems in determining platelets in subjects with macrothrombocytopenias, but they also suggest the severity of the defect is greatly variable.

The limitations of some instruments used in our study explain why, in the whole patient population, the sensitivity of the cut-off value for MPV of more than 51% with respect to controls was much lower than that obtained in the previous investigation that used an ADVIA 120 analyser (65% *versus* 83%) (Noris *et al*, 2009). However, considering only data obtained by the Abbott Cell-Dyn 3700 instrument in Centre 1, which examined the vast majority of patients with ITs enrolled in this study, the sensitivity rose to 83%, a value identical to that obtained in the prior study. The specificity of the cut-off value of MPV obtained in present study was similar to that obtained previously (91% *versus* 89%), although, as already discussed in the Results section, it is possible that this figure was a little overestimated. Thus, the diagnostic accuracy of the MPV cut-off value largely depends on the instrument used and is high when a suitable counter is used. Conversely, the diagnostic accuracy of the MPV in distinguishing ITP from the non-macrocytic forms of inherited thrombocytopenias we examined seems to be unsatisfactory.

MPDs obtained by blood film evaluation classified ITs similarly to MPV concerning platelet size, with the only exception of *ANKRD26*-RT, which had platelets of normal size based on MPV and slightly enlarged at MPD evaluation. However, the specificity and sensitivity of the cut-off value of 3·3 μm were poor in distinguishing inherited macrothrombocytopenias from ITP (67% and 75% respectively) and were much lower than those obtained by Noris *et al* (2009) (88% and 89% respectively). The reason why MPD was less effective than MPV in this respect is unknown, since these two parameters are obviously strictly related. However, MPV is measured in platelet suspended in plasma, while MPD is evaluated in platelets adhered to glass slides, and this could make the difference in that it has been shown that the shape change occurring in *MYH9*-RD and BSS platelets during adhesion to glass differs from that of normal platelets (Milton *et al*, 1985). Moreover, MPV, unlike MPD, is measured in anticoagulated blood and we cannot exclude that the anticoagulant affects platelet size differently in different disorders. We have also to consider that the microscope measurement of MPD is a rather inaccurate test because it is based on the analysis of a limited number of platelets and is, at least partially, operator-dependent.

In conclusion, our study showed that platelet size evaluation is a useful tool for distinguishing inherited macrothrombocytopenias from ITP and that the better differentiation is obtained by the use of the MPV values given by appropriate cell counters. Inappropriate instruments can be identified either by comparison of platelet counts measured by the counter with those obtained by manual counting or by the evaluation of peripheral blood films, which show very large platelets in subjects with normal or only slightly enlarged MPV. From a practical point of view, we suggest that microscope evaluation of blood films and measurement of MPV by cell counters should be used in combination. Blood film examination enables the easy identification of very large platelets, which suggest a diagnosis of inherited macrothrombocytopenias. If very large platelets are not identified, a MPV increased by more than 51% with respect to controls represents an important element that supports the suspicion of a hereditary macrothrombocytopenia.
